# Abdominal Clues From a Neurotropic Virus: An Atypical West Nile Case Caught During a Fever of Unknown Origin Workup

**DOI:** 10.7759/cureus.85760

**Published:** 2025-06-11

**Authors:** Archa Roy, Vikash A Ramtahal, Yan Naing Tun, Kantash Kumar

**Affiliations:** 1 Internal Medicine, Maimonides Medical Center, New York City, USA

**Keywords:** abdominal symptoms, colitis, encephalitis, fever of unknown origin, west nile virus

## Abstract

West Nile virus (WNV) is a mosquito-borne ribonucleic acid (RNA) virus usually transmitted by *Culex *mosquitoes. While most infections are asymptomatic, WNV can cause severe neuroinvasive disease, including meningitis and encephalitis, with rare cases of seizures and strokes. Diagnosis typically involves detecting WNV-specific immunoglobulin M (IgM) antibodies or viral RNA. Management is supportive.

This report details an unusual WNV presentation in a 50-year-old male patient with chronic lymphocytic leukemia (CLL) who experienced gastrointestinal symptoms and persistent high-grade fevers in early fall. This atypical presentation initially led to a diagnosis of pyrexia of unknown origin (PUO), delaying proper identification. The case highlights the importance of considering arboviral infections like WNV, especially during peak transmission months (July-September), to facilitate prompt diagnosis, reduce unnecessary investigations, and improve healthcare efficiency.

## Introduction

West Nile virus (WNV) is a mosquito-borne, single-stranded ribonucleic acid (RNA) virus belonging to the *Flaviviridae *family. Since its first appearance in the United States during a 1999 outbreak in New York City, WNV has become endemic across the contiguous United States. The virus is primarily transmitted through the bite of infected *Culex *mosquitoes, although rare cases have been documented through blood transfusion and organ transplantation. According to the Centers for Disease Control and Prevention (CDC), more than 16,000 cases of neuroinvasive disease and over 1,500 associated deaths have been reported since 1999, with an estimated 780,000 total infections having occurred [1, 2]. The highest incidence is noted in the Midwest during the peak transmission period from mid-July to early September [2].

Clinical manifestations of WNV infection vary widely, ranging from asymptomatic or mild febrile illness to severe neuroinvasive disease such as meningitis, encephalitis, and acute flaccid paralysis. Immunocompromised individuals, including those with hematologic malignancies such as chronic lymphocytic leukemia (CLL), are particularly vulnerable to severe or atypical presentations. In some instances, patients may present with nonspecific symptoms such as persistent fever and undergo extensive workup for pyrexia of unknown origin (PUO) before a diagnosis is made [3].

Here, we report a case of a 50-year-old male patient with CLL who presented with gastrointestinal symptoms (abdominal pain, nausea) and persistent high-grade fevers in early fall. The initial clinical picture and imaging findings suggested enteritis, delaying recognition of the underlying viral etiology. The eventual diagnosis of West Nile virus infection was confirmed via cerebrospinal fluid (CSF) immunoglobulin M (IgM) serology. This case highlights the importance of maintaining a high index of suspicion for arboviral infections like WNV, especially during peak transmission months, in order to facilitate timely diagnosis, reduce unnecessary investigations, and optimize healthcare resource utilization.

## Case presentation

A 50-year-old male patient with a medical history of CLL on active chemotherapy treatment presented in early fall with a three-day history of progressively worsening intense epigastric and right lower quadrant abdominal pain accompanied by nausea and diarrhea. He reported no recent travel outside of New York City and no history of recent blood transfusions or organ transplantation. Notably, rash, lymphadenopathy, and hepatosplenomegaly were absent on examination. Examination of the neurological system was normal without any meningeal signs.

On physical exam, the patient was alert and oriented and noted pyrexia with chills and rigor. Abdominal examination demonstrated epigastric and lower abdominal tenderness. Initial laboratory results showed an elevated white blood cell (WBC) count of 19,000/µL. Given the constellation of abdominal symptoms, infectious colitis was the primary differential diagnosis.

Further workup included gastrointestinal polymerase chain reaction (PCR),* Clostridioides difficile* toxin assay, urinalysis, urine culture, respiratory viral panel, and blood cultures (×2 sets), all of which were negative. Serologic testing for HIV and Venereal Disease Research Laboratory (VDRL) and a hepatitis panel were also non-reactive. Inflammatory markers, including erythrocyte sedimentation rate (ESR), C-reactive protein (CRP), and procalcitonin, were within normal limits.

**Figure 1:**
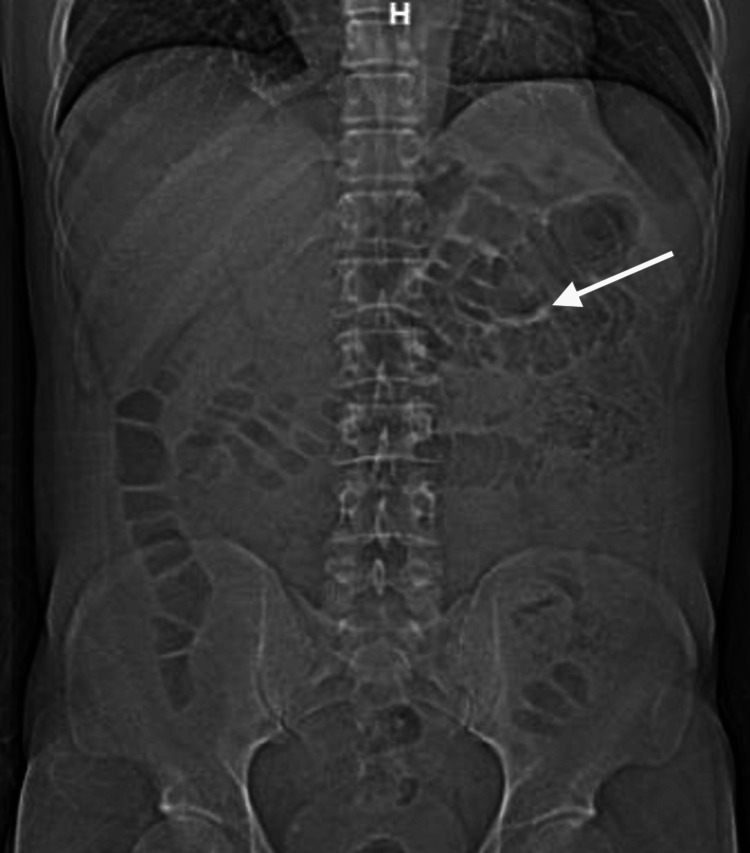
A CT scan of the abdomen and pelvis (anteroposterior (AP)) view showing bowel wall thickening suggestive of ileocolitis

A computed tomography (CT) scan of the chest, abdomen, and pelvis showed multiple prominent fluid- and air-filled loops of small bowel, thickening of the distal esophagus, with mild fatty infiltration of the terminal ileum, suggestive of possible inflammation (Figure 1). These findings supported the working diagnosis of colitis, and the patient was started on broad-spectrum empiric antimicrobial therapy with meropenem, vancomycin, and acyclovir due to his immunocompromised status.

Oncology was consulted early in the hospital course to evaluate for possible Richter’s transformation given the persistent fevers and elevated WBC count; however, the absence of lymphadenopathy, hepatosplenomegaly, or other typical clinical signs, along with diagnostic imaging and laboratory studies, ruled out this possibility.

Despite antibiotic therapy, the patient started to have altered mental status and continued to have high-grade fevers, reaching up to 106° Fahrenheit (F) without any meningeal signs. This prompted further evaluation for fever of unknown origin, leading to a lumbar puncture. CSF analysis revealed an opening pressure of 17 cm H₂O, elevated protein (126 mg/dL), and lymphocytic-predominant pleocytosis (55%). Electroencephalogram demonstrated global cerebral dysfunction with slow wave spikes and no epileptiform changes.

**Figure 2:**
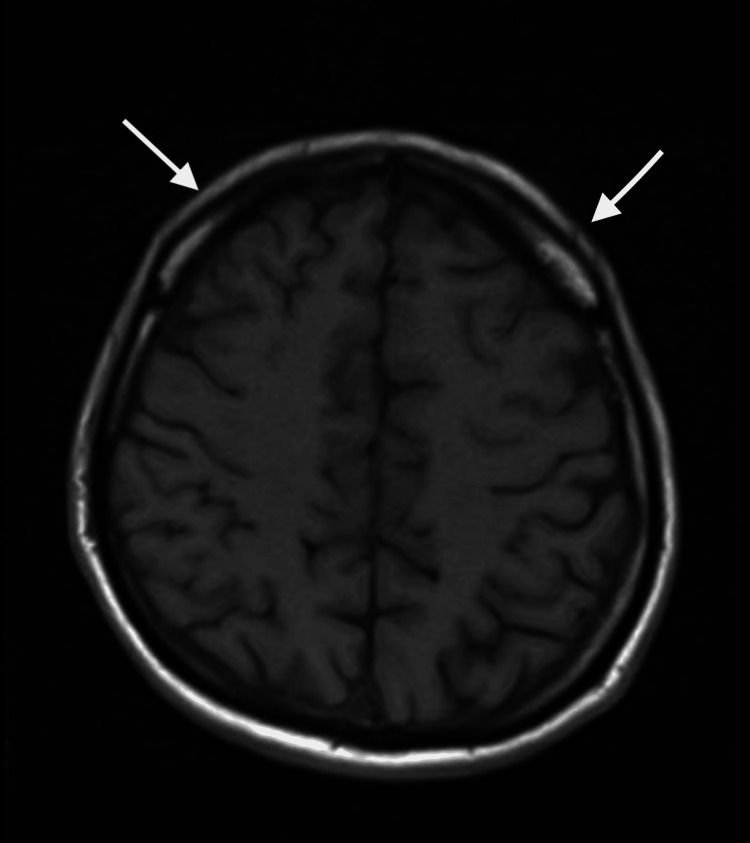
MRI of the brain showing subarachnoid punctate hyperintensity

Magnetic resonance imaging (MRI) of the brain showed scattered punctate hyperintensities in the subarachnoid space on post-gadolinium contrast transverse relaxation time (T2) image, which were interpreted as likely artefactual, with no definitive evidence of leptomeningeal involvement (Figure 2).

The Mayo Exome Sequencing-2 (ES2) panel for autoimmune and paraneoplastic encephalitis was negative, which is used to detect autoantibodies.

CSF serology eventually returned positive for WNV IgM, confirming a diagnosis of neuroinvasive WNV infection. The delay in diagnosis was attributed to the initial focus on gastrointestinal causes, particularly colitis, which diverted attention from a central nervous system etiology during the early phase of workup. Additional investigations, including peripheral smear, creatine phosphokinase, repeated HIV testing, autoimmune panel, and repeat blood cultures, remained unremarkable or negative (Table 1). The patient was eventually diagnosed with WNV encephalitis. With supportive care, the patient had a good clinical response, and his neurological symptoms resolved over the course of two months.

**Table 1:** Significant laboratory findings CSF: cerebrospinal fluid; IgM: immunoglobin M

Test	Result	Normal reference range
White blood cell (WBC) count	19,000 /µL	4,000 – 11,000 /µL
Ferritin	338 ng/mL	12 – 300 ng/mL
Lactate dehydrogenase (LDH)	264 U/L	140 – 280 U/L
CSF opening pressure	17 cm H₂O	10 – 20 cm H₂O
CSF protein	126 mg/dL	15 – 45 mg/dL
CSF cell count	Lymphocytic pleocytosis-55%	0 – 5 WBCs/µL, mostly lymphocytes
CSF West Nile virus IgM	Positive	Negative

## Discussion

WNV, a single-stranded, positive-sense RNA virus of the *Flaviviridae *family, is primarily transmitted through the bite of infected *Culex *mosquitoes. While the majority of infected individuals remain asymptomatic or experience a mild febrile illness, approximately 1% develop neuroinvasive disease, including meningitis, encephalitis, or acute flaccid paralysis. This is more likely to occur in immunocompromised individuals and the elderly [4,5]. Our patient, a 50-year-old male patient with CLL, represents a classic example of how an immunocompromised state can predispose to an atypical and severe presentation of WNV.

Since its first appearance in the Western Hemisphere during the 1999 outbreak in New York City, WNV has become endemic throughout the continental United States [6]. According to the CDC, 1,466 cases of WNV were reported in 2024, reflecting continued annual variation. Historically, 89% to 93% of WNV cases occur during the peak transmission months of July through September, with the highest incidence reported in the Midwest and West North Central states. These seasonal and regional patterns underscore the importance of maintaining a high index of suspicion for WNV during late summer and early fall, especially in high-risk populations [7,8].

Neuroinvasive disease typically presents with a constellation of fever, altered mental status, meningeal signs, and focal neurological deficits. Acute flaccid paralysis may mimic poliomyelitis and carry the risk of respiratory compromise. While gastrointestinal symptoms such as abdominal pain, nausea, and diarrhea are extremely rare in WNV encephalitis, our patient presented with this atypical manifestation, which initially led to a diagnostic focus on gastrointestinal pathology and a prolonged workup for PUO [7-9].

CSF analysis remains the cornerstone of WNV diagnosis in suspected neuro-invasive disease. The typical CSF profile includes lymphocytic pleocytosis, elevated protein, and normal glucose levels. The most specific diagnostic modality is detection of WNV-specific IgM antibodies in CSF. As IgM does not cross the blood-brain barrier, its presence in CSF confirms central nervous system involvement. In contrast, serum IgM is less specific due to potential cross-reactivity with other flaviviruses. Nucleic acid amplification testing (NAAT) for WNV RNA can be useful, particularly in immunocompromised hosts or early in the disease course, though it has lower sensitivity in immunocompetent patients [10].

Neuroimaging findings in WNV encephalitis, such as T2/fluid-attenuated inversion recovery (FLAIR) hyperintensities in the basal ganglia, thalamus, or brainstem, can support the diagnosis but are not specific. In our case, these typical features were absent on MRI, likely due to poor patient cooperation during altered mental status. This absence further complicated the diagnostic process. Treatment of WNV remains supportive. Interventions focus on managing complications such as airway protection, seizure control, and secondary infections. No antiviral therapy has demonstrated a proven benefit in clinical trials. The Infectious Diseases Society of America (IDSA) recommends using acyclovir empirically until herpes simplex virus (HSV) is excluded, but it does not endorse routine antiviral or immunomodulatory treatment for WNV [11]. Similarly, intravenous immunoglobulin (IVIG) and corticosteroids have not shown consistent clinical benefit in randomized studies [12-15].

This case highlights the importance of considering WNV in patients with persistent fever during peak transmission months, particularly in those who are immunocompromised. Atypical presentations, such as gastrointestinal symptoms, can make diagnosis challenging and may lead to broad diagnostic workups. Early recognition of seasonal and geographic patterns of WNV can help guide more focused testing, potentially reducing unnecessary investigations, hospital stays, and healthcare costs. While treatment is supportive, timely diagnosis can improve clinical efficiency and patient care by avoiding delays and streamlining resource use.

## Conclusions

WNV should remain a key differential in patients presenting with persistent fever during the peak transmission season, especially those with underlying immunosuppression. This case underscores how atypical presentations can obscure the diagnosis, leading to extensive and costly workups. Awareness of the seasonal epidemiology and clinical variability of WNV can aid in earlier identification, allowing for targeted testing, reducing unnecessary investigations, and improving overall healthcare efficiency, even when definitive treatment remains supportive.
